# Miscellaneous Information

**Published:** 1862-10

**Authors:** 


STIMULANTS AND TONICS.—Chew a pinch or two of green tea when exhausted, or when on guard, or when on special hard duty, and repeat every half-hour, more or less; it enlivens without the subsequent debility of spirits. *Cayenne Pepper*, called "capsicum," acts similarly; a pinch at a time will modify that excessive weariness or sleepiness, and is far more powerful than tea, in all its good effects; while its convenience for carriage in point of bulk, renders it the most valuable substance that a soldier can carry, as to nourishment, thirst, or invigorating powers. A single pinch in a cup of "flat" water will make it quite palatable. A third of a tea-spoonful taken at meals, morning, noon, and night, with the food or drink, not only invigorates digestion, but is a great antagonizer of dyspeptic and all bowel complaints in armies.

STOCKINGS.—The feet will be blistered by a six hours' march in cotton stockings; wear woolen, rubbing the soles well with tallow or soap, if you can, when a heavy march is in prospect.

FOOD.—One pound of sugar mixed with three pounds of ground wheat or corn (with the bran) called "*Pinole*," is one of the most nutritious and healthy articles of food in the world for an army, and is easily carried. Jerked beef is next, made by cutting fresh beef in strips, and drying them in the sun, with as little salt as possible; it will keep good a year.

ARMY BEVERAGES.—Col. Dawes, an experienced East-Indian officer, says that coffee and tea should take the place of liquors, and that every man should have some as soon as he gets up in the morning, and also at sun-down. During the Crimean war, it was found that when the soldiers obtained warm coffee, they sustained fatigue and were comparatively healthy; but when they were in the trenches, and could not get good warm tea or coffee, they were subject to dysentery or bloody flux.

GUNNERS may avoid more or less permanent deafness by putting into their ears before an action a bit of wool or cotton dipped into a mixture of forty grains of belladonna with one ounce of glycerine—keep it in until next morning.

DIRECTIONS TO ARMY-SURGEONS ON THE FIELD OF BATTLE.—The following, taken from Mr. G. J. Guthrie's pamphlet on the Hospital Brigade, is copied from the London *Lancet*. Mr. Guthrie was Surgeon-General to the British forces during the Crimean war, and consequently speaks from extensive opportunities of observation:

1. Water being of the utmost importance to wounded men, care should be taken, when before the enemy, not only that the barrels attached to the conveyance-carts are properly filled with good water, but that skins for holding water, or such other means as are commonly used in the country for carrying it, should be procured and duly filled.

2. Bandages or rollers, applied on the field of battle, are, in general, so many things wasted, as they become dirty and stiff, and are usually cut away and destroyed, without having been really useful; they are therefore not forthcoming when required, and would be of no use.

3. Simple gun-shot wounds require nothing more for the first two or three days than the application of a piece of wet or oiled linen, fastened on with a strip of sticking-plaster, or, if possible, kept constantly wet and cold with water. When cold disagrees, warm water should be substituted.

4. Wounds made by swords, sabers, or other sharp-cutting instruments, are to be treated principally by position. Thus, a cut down to the bone, across the thick part of the arm, immediately below the shoulder, is to be treated by raising the arm to or above a right angle with the body, in which position it is to be retained, however inconvenient it may be. Ligatures may be inserted, but through the skin only. If the throat be cut across in front, any great vessels should be tied, and the oozing stopped by a sponge. After a few hours, when the oozing is arrested, the sponge should be removed, and the head brought down toward the chest, and retained in that position without ligatures; if this is done too soon, the sufferer may possibly be suffocated by the infiltration of blood into the areolar tissue of the parts adjacent.

5. If the cavity of the chest is opened into by a sword or lance, it is of the utmost importance that the wound in the skin should be effectively closed, and this can only be done by sewing it up as a tailor or lady would sew up a seam, skin only being included; a compress of lint should be applied over the stitches, fastened on by sticking-plaster. The patient is then to be placed on the wounded side, that the lung may fall down, if it can, upon or apply itself to the wounded part, and adhere to it, by which happy and hoped-for accident life will in all probability be preserved. If the lung should be seen protruding in the wound, it should not be returned beyond the level of the ribs, but be covered over by the external parts.

6. It is advisable to encourage previously the discharge of blood from the cavity of the chest, if any have fallen into it; but if the bleeding from within should continue, so as to place the life of the sufferer in danger, the external wound should be closed, and events awaited.

7. When it is doubtful whether the bleeding proceeds from the cavity of the chest or from the intercostal artery, (a surgical bug-bear,) an incision through the skin and the external intercostal muscle will expose the artery close to the edge of the rib, having the internal intercostal muscle behind it. The vessel thus exposed may be tied, or the end pinched by the forceps, until it ceases to bleed. Tying a string round the ribs is a destructive piece of cruelty; and the plugs, etc., formerly recommended, may be considered as surgical incongruities.

8. A gun-shot wound in the chest can not close by adhesion, and must remain open. The position of the sufferer should therefore be that which is most comfortable to him. A small hole penetrating the cavity is more dangerous than a large one, and the wound is less dangerous if the ball goes through the body. The wounds should be examined, and enlarged if necessary, in order to remove all extraneous substances, even if they should be seen to stick on the surface of the lungs; the opening should be covered with soft oiled or wet lint—a bandage when agreeable. The ear of the surgeon and the stethoscope are invaluable aids, and ought always to be in use; indeed, no injury of the chest can be scientifically treated without them.

9. Incised and gun-shot wounds of the abdomen are to be treated in *nearly* a similar manner; the position in both being that which is most agreeable to the patient, the parts being relaxed.

10. In wounds of the bladder, an elastic catheter is generally necessary. If it can not be passed, an opening should be made in the perinæum for the evacuation of the urine, with as little delay as possible.

11. In gun-shot fractures of the skull, the loose broken pieces of bone, and all extraneous substances, are to be removed as soon as possible, and depressed fractures of bone are to be raised. A deep cut, made by a heavy sword, through the bone into the brain, generally causes a considerable depression of the inner table of the bone, whilst the outer may appear to be merely divided.

12. An arm is rarely to be amputated, except from the effects of a cannon-shot. The head of the bone is to be sawn off, if necessary. The elbow-joint is to be cut out, if destroyed, and the sufferer, in either case, may have a very useful arm.

13. In a case of gun-shot fracture of the upper arm, in which the bone is much splintered, incisions are to be made for the removal of all the broken pieces which it is feasible to take away; the elbow is to be supported; the fore-arm is to be treated in a similar manner; the splints used should be solid.

14. The hand is never to be amputated unless all or nearly all its parts are destroyed. Different bones of it and of the wrist are to be removed when irrecoverably injured, with or without the metacarpal bones and fingers, or the thumb; but a thumb and one finger should always be preserved when possible.

15. The head of the thigh-bone should be sawn off when broken by a musket-ball. Amputation at the hip-joint should only be done when the fracture extends some distance into the shaft, or the limb is destroyed by cannon-shot.

16. The knee-joint should be cut out when irrecoverably injured; but the limb is not to be amputated until it can not be avoided.

17. A gun-shot fracture of the middle of the thigh, attended by great splintering, is a case for amputation. In less difficult cases, the splinters should be removed by incisions, particularly when they can be made on the upper and outer side of the thigh. The limb should be placed on straight, firm splint. A broken thigh does not admit of much, and sometimes of no extension, without an unadvisable increase of suffering. An inch or two of shortening in the thigh does not so materially interfere with progression as to make the sufferer regret having escaped amputation.

18. A leg injured below the knee should rarely be amputated in the first instance, unless from the effects of a cannon-shot. The splinters of bone are all to be immediately removed by saw or forceps, after due incisions. The limb should be placed in iron splints, and hung on a permanent frame, as affording the greatest comfort and probable chance of ultimate success.

19. An ankle-joint is to be cut out unless the tendons around are too much injured, and so are the tarsal and metatarsal bones and toes. Incisions have hitherto been too little employed in the early treatment of these injuries of the foot, for the removal of extraneous substances.

20. A wound of the principal artery of the thigh, in addition to a gun-shot fracture, renders immediate amputation necessary. In *no other part* of the body is amputation to be done in the first instance for such injury. Ligatures are to be placed on the wounded artery, one above, the other below the wound, and events awaited.

21. The occurrence of mortification in any of these cases will be known by the change of color in the skin. It will rarely occur in the upper extremity, but will frequently do so in the lower. When about to take place, the color of the skin of the foot changes from the natural flesh color to a tallowy or mottled white. Amputation should be performed immediately above the fractured part. The mortification is yet local.

22. When this discoloration has not been observed, and the part shrinks, or gangrene has set in with more marked appearances, but yet seems to have *stopped* at the ankle, delay is, perhaps, admissible; but if it should again spread, or its cessation be doubtful, amputation should take place forthwith, although under less favorable circumstances. The mortification is becoming or has become constitutional.

23. Bleeding, to the loss of life, is not a common occurrence in gun-shot wounds, although many do bleed considerably, seldom, however, requiring the application of a tourniquet as a matter of necessity, although frequently as one of precaution.

24. When the great artery of the thigh is wounded, (not torn across,) the bone being *uninjured*, the sufferer will probably bleed to death, unless aid be afforded, by making compression above and on the bleeding part. A long but not broad stone tied sharply on with a handkerchief, will often suffice until assistance can be obtained, when both ends of the divided or wounded artery are to be secured by ligatures.

25. The upper end of the great artery of the thigh bleeds scarlet blood; the lower end dark venous-colored blood; and this is not departed from in a case of accidental injury, unless there have been previous disease in the limb. A knowledge of this fact or circumstance, which continues for several days, will prevent a mistake at the moment of injury, and at a subsequent period, if secondary hemorrhage should occur. In the *upper* extremity both ends of the principal artery bleed scarlet blood, from the free collateral circulation, and from the anastomoses in the hand.

26. From this cause, mortification rarely takes place after a wound of the principal artery of the arm, or even of the arm-pit. It *frequently* follows a wound of the principal artery in the upper, middle, or even lower parts of the thigh, rendering amputation necessary.

27. It is a great question, when the bone is *uninjured*, where and at what part the amputation should be performed. Mortification of the foot and leg, from such a wound, is disposed to stop a little below the knee, if it should not destroy the sufferer; and the operation, if done in the first instance, as soon as the tallowy or mottled appearance of the foot is observed, should be done at that part; the wound of the artery and the operation for securing the vessel above and below the wound being left unheeded. By this proceeding, when successful, the knee-joint is saved, whilst an amputation above the middle of the thigh is always very doubtful in its results.

28. When mortification has taken place from any cause, and has been arrested below the knee, and the dead parts show some sign of separation, it is usual to amputate above the knee. But not doing it, but by gradually separating and removing the dead parts, under the use of disinfecting medicaments and fresh air, a good stump may be ultimately made, the knee-joint and life being preserved, which latter is frequently lost, after amputation, under such circumstances.

29. Hospital gangrene, when it unfortunately occurs, should be considered to be contagious and infectious, and is to be treated locally by destructive remedies, such as nitric acid, and the bivouacking or encamping of the remainder of the wounded, if it can be effected, or their removal to the open air.

30. Poultices have been very often applied in gun-shot wounds, from laziness, or to cover neglect, and should be used as seldom as possible.

31. Chloroform may be administered in all cases of amputation of the upper extremity and below the knee, and in all minor operations; which cases may also be deferred, without disadvantage, until the more serious operations are performed.

32. Amputations of the upper and middle parts of the thigh are to be done as soon as possible after the receipt of the injury. The administration of chloroform in them, when there is much prostration, is doubtful, and must be attended to, and observed with great care—the question whether it should or should not be administered in such cases being undecided.

No surgeon is truly fit for his place, however scientific and skillful, who has not the tact to encourage and sympathize with the sick and wounded soldier; a word, a look, a gesture may so wake up the nervous activities, and the moral sentiments of hope, ambition, determination, or patriotism, that the system will rise superior to disease and cast it off in an hour; when, on the other hand, a want of sympathy on the part of the medical attendant, a mere mechanical or routine attention to the objects in hand, would have allowed the invalid to pass into the grave. There is no incompatibility between firmness and kindness; and that surgeon is most a man who makes the wisest combination of these two prime qualities of the intellect and of the heart. Each soldier is at this juncture a part of his country's hope, and, although but a unit in himself, he merits, in a disablement brought upon him in the discharge of his duty to the nation, a consideration and a tenderness in the hour of his suffering, which a man will always give, and which is withheld only by the brute.

To show how inevitably a soldier's gratitude wells upward toward those who minister to his wants it is only necessary to recall the beautiful fact that when Florence Nightingale passed along the halls of the Crimean hospitals, the men who could not go to greet her would crawl to the side of their beds and kiss her shadow as she passed.

There are many things which a wounded soldier may do for himself, and many others which one who is well may do for his comrade which will prevent an immense amount of suffering and may save life itself, by gaining time or by keeping things in *statu quo*, from progressing, until a surgeon can be had. Hence, to know these things beforehand is to make one a life-saver.

Very red blood, sparkling, bright, thin, is from the arteries, and the man will soon die if it flows fast, because it is the life of the body and flows directly from the heart. If the wound is on the back of the hand, or face, or scalp, or on any part of the body where there is not much more than skin and bone, press the thumb tighter and tighter above the wound, that is, between the wound and the heart, and continue the compression until the blood ceases to flow and until the surgeon arrives. If a limb is bleeding, or a part where there is more flesh, tie a knot in the center of a handkerchief, and let that knot rest over the bleeding vessel above the wound, then bring the ends of the handkerchief around the limb, and tie it as tight as possible; if the artery is a very large one, there is not time for this, or it might not be efficient, the handkerchief must be tied loosely around the limb, above the wound, a stick put in between the skin and the handkerchief, and twisted around until the bleeding ceases, as named on page eight. Or, if you can see the orifice of a bleeding artery, make a hook of a needle by heating it red hot, cool it, then hook it into the artery, draw it out, or it may be drawn out with a pair of tweezers, pincers, or tongs, then tie a strings tightly around the end of the bleeding vessel and let it remain until the surgeon arrives. Profuse bleedings about the head or face, or other bony parts, may be arrested if from small blood-vessels or mere flesh-wounds, by compresses of linen or cotton kept wet with cold water, or by lint, or by cobwebs. Never apply any thing to a wound which irritates, but wash it with something that is soothing and healing, such as castile soap. If a bone is broken, keep still, bind up the parts, and apply cold water most freely until the surgeon arrives.

Poisonous Bites, Stings, etc.—If there is no sore about the mouth or lips, suck it out and spit it out, for some minutes in succession, or, better still, wash the part most freely with spirits of hartshorn, because that is an alkali, and the bites and stings of reptiles and insects are acid, hence are nullified by the strong alkali. If there is no hartshorn at hand, make an alkali by wetting fresh wood-ashes with water and apply it to the wound as a poultice, renewing it every half-hour until relieved. Strong alkaline washes instantly applied are believed a perfect preventive of serious harm from all bites and stings, except from a mad dog or other rabid creature. Insects are removed from the nose and ears by spirting in water from the mouth; sweet oil with a syringe is as good, if at hand.

Soldier Items.—*Swallowing Poison.*—Stir in a glass of water a heaping teaspoonful each of salt and kitchen mustard, and drink it instantly—this will empty the stomach in a minute. To antagonize any poison that may be left, swallow the whites of two or three eggs; then drink a cup or two of very strong coffee, or as much sweet milk or cream, if impossible to get coffee.

Poisoned Vines.—Apply a paste made of gunpowder, or sulphur, with milk; renew night and morning, until cured. Live on gruel, soups, rice, and other mild food, having the bowels to act twice a day.

Signs of Death.—Bury no man unless his head is off, or the abdomen begins to turn green or dark, the only sure signs, but always sure, of actual death. If there is haste, cut off a toe or finger, which would wake up the slightest spark of life left.

To Stop Bleeding.—Four or five drops of Perchlorid of Iron will check completely the flow of blood from all except the largest arteries; half a teaspoonful will arrest even their bleeding. Each non-commissioned officer should have two ounces of this in a flat tin bottle, wound around with a little cotton batting, on a bit of which the liquid could be dropped for application.

Obedience is not servility, it is a high duty; it is not cowardly, but proudly honorable in a soldier. If your officer speaks sharply, it is neither to insult nor to browbeat; it is to wake up attention, instant and implicit.

For every wounded soldier taken to the hospital in the Crimean war, twelve were taken on account of disease; disease which could be avoided in more than half the cases by such care as the soldier can take of himself, as directed in these pages. Of the 15,000 lives lost in the Mexican war, only 1548 were from battle. The United States Sanative Commission report that 104 soldiers became sick to each 1000 in the present war.

Shirts.—A distinguished British Army Surgeon says: More than one half of all army diseases in warm countries are owing to the exposure of the abdomen to changes of temperature. Shirts should reach the thigh.

Inner Clothing.—Every garment which touches a soldier's skin should be woolen in all seasons, most important in the warmest weather. It is impossible to over-estimate the value of this one item to the health of an army.

Limestone-Water.—One teaspoonful of vinegar, in a pint of such water, will antagonize all its ill effects on the bowels of those unaccustomed to it.

Dirty Water.—As much powdered alum as will rest on a dime, stirred in a pail of water, will clarify it in five minutes.

Saving Life.—In the first seven months of the Crimean campaign, the soldiers died at the rate of 60 out of a 100 per annum, while for the last five months of the war not so many soldiers died of disease as at home, owing to a more systematic and rigid attention to five things: 1st. Selecting healthful camps; 2d. Enforcing strict cleanliness; 3d. Avoiding *unnecessary* exposure; 4th. Proper preparation of healthful food; 5th. Judicious nursing.

A True Soldier is considered one of the highest types of a man. But that officer merits not the name or the title he bears, who does not make the comfort and health of his men a subject of unceasing thought, and of the most indefatigable effort.

Camp-Grounds.—An elevation is a hundred-fold better than a flat or a hollow; open ground better than among trees; better for health, safer from surprise, and stronger for attack and defense, even if it is calculated to stay but a few hours. Let the tent face the south, the top screened with brush-wood, and if practicable with a floor of boards three inches above the ground, and a ditch around the tent six or eight inches deep.

DRINKING WATER improperly has killed thousands of soldiers. If possible, avoid drinking any thing on a march. If you must drink, the colder the water the less will it satisfy thirst. ☞ Half a glass of water drank in sips, swallowing each sip, with a few seconds interval, will more effectually satisfy thirst, and that without any danger, than a quart taken in the usual manner at one draught. It is greatly safest, *while marching*, to rinse the mouth only, but do that to the utmost extent desired, spirting out the water as soon as it becomes warm. Chewing even a stick or pebble moderates thirst.

MITTENS, for cold weather, should have a thumb and one finger, the other three fingers together, so as to use the trigger handily.

BOWEL AFFECTIONS are said to be cured, if at all curable, by drinking from one half to four half pints of a tea made of the inner bark of the sweet-gum tree, boiled until of the taste and color of strong coffee, with or without sugar, cold or hot. The tree abounds southward.

CROMWELL'S DISCHARGED SOLDIERS.—Immorality and irreligion are among the great evils of war. Knowing this, every Christian should be most diligent, not only in prayer for the soldiers, and in furnishing them with religious privileges in the camp, but in cherishing a strong and enlightened public religious sentiment. Public sentiment is a powerful stimulant to moral principle, as well as to patriotic feeling. It hence becomes the whole Christian community to frown upon Sabbath-day parades and displays. A country sometimes suffers immensely after a war is over, from the murders, robberies, thefts, and other depredations and immoralities of its own discharged soldiers. The principles and habits of the camp follow, or rather accompany, the men through life. In this aspect of the case, it becomes not only Christians who feel for men's immortal welfare, but it becomes all who have personal interests at state, all who have property or families to preserve, to see to the character of the camp. Cromwell kept up religion in his army. He had chaplains, prayers, Sabbaths, preaching, Bibles, psalm-books, and withal the bravest men that ever went into battle. And after their return to private life, history, in recording their heroic deeds, bears this testimony to their moral worth: Fifty thousand men, accustomed to the profession of arms, were at once thrown on the world. In a few months there remained not a track indicating that the most formidable army in the world had been absorbed into the mass of the community. The royalists themselves confessed that in every department of honest industry, the discarded warrior prospered beyond other men, that none was charged with any theft or robbery, that none was heard to ask an alms, and that, if a baker, a mason, or a wagoner attracted notice by his diligence and sobriety, he was in all probability one of Oliver's old soldiers.

OBSERVANCE OF THE SABBATH.—*General Order No.* 7.—The Major-General Commanding desires and requests that in future there may be a more perfect respect for the Sabbath on the part of his command. We are fighting in a holy cause, and should endeavor to deserve the benign favor of the Creator. Unless in a case of an attack by the enemy, or some other extreme military necessity, it is commended to commanding officers that all work shall be suspended on the Sabbath; that no unnecessary movements shall be made on that day; that the men shall, as far as possible, be permitted to rest from their labors; that they shall attend divine service after the customary Sunday morning inspections, and that officers and men alike use their influence to insure the utmost decorum and quiet on that day. The General commanding regards this as no idle form. One day's rest in seven is necessary for man and animals. More than this, the observance of the holy day of the God of mercy and of battles is our sacred duty. WASHINGTON, Sept. 6th, 1861.

